# Supplementary material for: Specific Mycobacterium tuberculosis Strain Circulating in Prison Revealed by Cost-Effective Amplicon Sequencing
Source: Microorganisms. 2024 May 15;12(5):999. doi: 10.3390/microorganisms12050999 (PMC11123834; doi:10.3390/microorganisms12050999)
Supplement: Supplementary file 1 [file microorganisms-12-00999-s001.zip › microorganisms-2927840-supplementary.pdf]

microorganisms

MDPI

Supplementary Material

File S1. Pipeline used to analyze sequencing data starting from fastq generated after Illumina run:

- Sickle (sequence quality filter)

```
sickle pe -f <name> _R1.fastq -r <name>_R2.fastq -t sanger -o <name>_F_trimmed -p <name>_R_trimmed -s <name>_trimmed
```

- BWA (mapping against reference genome H37v_NC_000962.3)

```
bwa index -p H37Rv_NC_000962.3_index -a is H37Rv_NC_000962.3.fasta

bwa mem / H37Rv_NC_000962.3_index ../<name>_R1.fastq ../<name>_R2.fastq > -o  <name>_alin_vs_lin.sam
```

- Sam to bam (transformation of bwa ouput to analize SNP)

```
samtools view -S -b <name>_alin_vs_lin.sam > <name>_alin_lin.bam

samtools sort -o <name>_sorted_alin_lin.bam <name>_alin_lin.bam

samtools index -b <name>_sorted_alin_lin.bam <name>sorted_alin_lin.bam.bai
```

- bcftools mpileup (SNPs analysis)

```
bcftools mpileup -O b -o <name>.bcf -f /gen_lineage.fasta <name>_sorted_alin_lin.bam
```

- Variant calling (Identification of SNPs)

```
bcftools call -o <name>.vcf -mv <name>.bcf

bcftools filter --exclude "QUAL < 30" <name>.vcf > <name>_filtered.vcf

bcftools view -v snps <name>_filtered.vcf | more

ls | less -S <name>.vcf | grep -v "##" | cut -f1,2,3,4,5,6,10 | sed '/^#/{x;p;x}' > snp.txt
```

Table S1: Sample information. Age, Gender and bacterial culture results, previous culture (relapse) and HIV information were recorded. Reclusion Center: N°4 (-34.8036113,-56.3547671), N°5 (-34.791844, -56.230891), N°6 (-34.8363927,-56.092903), N°7 (-34.5270384,-56.2663051), N°18 (-33.379260, -56.521940). * Detected at Entry Center, then transferred to N°4.

ID	Age	Sex	Bacterial culture	Relapse	HIV	Reclusion Center
1	25	M	Positive ++	0	0	N°4*
2	28	M	Positive ++			N°6
3	20	M	Positive +	0	0	N°4*
4	31	M	Positive ++			N°4*
5	35	M	Positive <20	0	0	N°4*
6	43	F	Positive <20	0	0	N°18
7	60	M	Positive +++	0	0	N°4*
8	24	M	Positive ++	0	0	N°4*
9	23	M	Positive +	0	0	N°4*
10	46	M	Positive <20	0	0	N°4*
11	54	M	Positive +	0	0	N°4*
12	33	M	Positive <20	0	0	N°4*
13	27	M	Positive +++	0	0	N°6
14	26	M	Positive <20	0	0	N°4
15	26	M	Positive ++	0	0	N°4*
16	30	M	Positive +			N°4*
17	22	M	Positive <20	0	0	N°4*
18	28	M	Positive +++	0	0	N°4*
19	40	M	Positive <20	0	0	N°4*
20	39	M	Positive <20	0	0	N°4*
21	73	M	Positive <20	1	0	N°4*
22	37	M	Positive +	0	0	N°4*
23	25	F	Positive <20	0	0	N°5
24	48	F	Positive <20			N°5
25	21	M	Positive +++	0	0	N°6
26	43	M	Positive <20	0	0	N°4*
27	46	M	Positive +++	0	0	N°4*
28	24	M	Positive ++	0	0	N°4
29	25	M	Positive +	0	0	N°4*
30	41	M	Positive <20	0	0	N°4*
31	32	M	Positive <20	1	0	N°7
32	31	F	Positive ++	0	0	N°5
33	27	M	Positive +	0	0	N°7
34	30	M	Positive <20			N°6
35	23	M	Positive +++	0	0	N°4*
36	35	M	Positive +++	0	0	N°4*
37	34	M	Positive <20	0	0	N°6
38	38	M	Positive +++	1	0	N°6
39	29	M	Positive <20	0	0	N°4*
40	36	M	Positive +++	0	0	N°4*

41	19	M	Positive <20	0	0	N°7
42	29	M	Positive <20	0	1	N°4*
43	41	M	Positive <20	0	0	N°4*
44	32	M	Positive +++	0	0	N°6
45	35	M	Positive +	0	0	N°4
46	31	M	Positive <20	1	0	N°4
47	39	M	Positive <20	0	0	N°4*

Table S2: Lineage Region information. The table indicates the lineage, the name or denomination it receives, the gene and the position of the mutation with respect to the reference genome. Additionally, the base change, the codon number where it occurs, is indicated. They are all silent substitutions so there is no amino acid change.

Lineage	*Sublineage*	*Position**	*Gen coord.*	*Allelic change*	*Codon number*	*Codon Change*	*AA change*	*ID Locus*	*Gen name*
lineage7	Lineage 7	1137518	543	G/A	181	AAC/AAT	N/N	Rv1018c	glmU
lineage6	Lineage 6	1816587	399	C/G	133	GTC/GTG	V/V	Rv1617	pykA
lineage5	Lineage 5	1799921	339	C/A	113	GGC/GGA	G/G	Rv1599	hisD
lineage4.4.1.1	S	355181	684	G/A	228	AAG/AAA	K/K	Rv0291	mycP3
lineage4.3	LAM	764995	1626	C/G	542	GCC/GCG	A/A	Rv0668	rpoC
lineage4.1.2.1	Haarlem	107794	195	C/T	65	GCC/GCT	A/A	Rv0098	fcoT
lineage4.1.1	X	514245	1077	C/T	359	GTG/GTA	V/V	Rv0425c	ctpH
lineage4	Lineage 4	931123	171	T/C	57	TAT/TAC	Y/Y	Rv0835	lpqQ
lineage3	Lineage 3	3273107	894	C/A	298	GCC/GCA	A/A	Rv2936	drrA
lineage2	Lineage 2	497491	810	G/A	270	GAC/GAT	D/D	Rv0411c	glnH
lineage1	Lineage 1	615938	1104	G/A	368	GAG/GAA	E/E	Rv0524	hemL

*According to the reference genome NC_000962.3

Table S3: Region information. Genes associated with resistance to anti-tuberculosis drugs

Gene	*Resistance*	*Position**
eis	Kanamycin	2714124-2715332
embB	Ethambutol	4246514-4249810
gyrA	Fluoroquinolones	7302-9818
gyrB	Fluoroquinolones	5240-7267
inhA	Isoniazid	1674202-1675011
katG	Isoniazid	2153889-2156111
pncA	Pyrazinamide	2288681-2289241
rpoB	Rifampicin	759807-763325
rrS	Streptomycin	1471846-1473382

*According to the reference genome NC_000962.3

Table S4. Primers designed for lineage pool

Gene	*Lineage*	*Fwd/Rev*	*Sequence 5´-3´*	*Amplicon size (bp)*
ctpH	X	Fwd	GGAAAAGTGCCTCGACTGC	374
		Rev	GTTTCGCGCTGATCCAAG	
mycP3	S	Fwd	TGACGGCTTCTCTGGTGTG	400
		Rev	GCCACCGACAACACGTAG	
fcoT	Haarlem	Fwd	GGTTCCGATCGCAGAGGA	320
		Rev	TTGATCGCCGGGAGTTGTAA	
rpoC	LAM	Fwd	CGCCGGAAATCATCGTCAAC	648
		Rev	TCGGTGGTCAGGTAGTACAG	
hemL	Lineage 1	Fwd	GAAGTGGTTGGTACGGAATC	372
		Rev	ATGGCATGAAAGAACGCTGG	
glnH	Lineage 2	Fwd	GATACAACGTGTTCCAGGTG	356
		Rev	GTCTACCTCGACGCCAAC	
drrA	Lineage 3	Fwd	TGAAGGATCTGGACGCTATC	201
		Rev	TCAGAGACTCGGTGGGATC	
lpqQ	Lineage 4	Fwd	GATCAGTCGTGTGTTGCTCC	269
		Rev	TACGGAACTCTGACCAGTCG	
hisD	Lineage 5	Fwd	TCTGGGAGAATGGTCTACGTG	435
		Rev	CACCACGTTCATCACCAC	
pykA	Lineage 6	Fwd	ACAAGGTCGCCTATGAGC	364
		Rev	GAGATCCTCGATGTCCTTC	
glmU	Lineage 7	Fwd	CTGGCATGTACGGTCTGG	212
		Rev	GATGATCCCTTCGGCTAC	
alkA	Bovis	Fwd	GGCCTTCGTCGATACCTG	732
		Rev	AATGGAATGTGCGTAGTGAC	

Table S5. Primers designed for resistance pool

Gen	*Resistance*	*Fwd/Rev*	*Sequence 5´-3´*	*Amplicon size (bp)*
eis	Kanamycin	Fwd	AAAGCCCGTCAGCCTAGC	1280
		Rev	ATCGCGTGATCCTTTGCC	
embB	Ethambutol	Fwd1	GGATCGGTGGAGCAGTACC	1281
		Rev1	GTTGTTGAACGGCATCCAC	
		Fwd2	TACCGCCGCATTCACACT	1080
		Rev2	GGCTGGTTGGGTTTCATCACG	
		Fwd3	GAGTCCTGGCATCAATGGTT	978
		Rev3	CCAACACCCCTGCGCCGAC	
gyrA	Fluoroquinolones	Fwd	AAACGAGGAATAGATGACAG	2588
		Rev	CACCCCGACTCCTAACACTC	
gyrB	Fluoroquinolones	Fwd	GGGTAAAAACGAGGCCAGA	2114
		Rev	CAGGGTTGCGTTAGACATCC	
inhA	Isoniazid	Fwd	GGCCACTGACACAACACAAG	825
		Rev	CAGCAGGACGGCATCAAATTG	
katG	Isoniazid	Fwd1	CGATCAACCCGAATCAGC	1141
		Rev1	GGTGGATCCGATCTATGAGC	
		Fwd2	GTCAGTGGCCAGCATCGTCG	1044
		Rev2	GATCACAGCCCGATAACACC	
pncA	Pyrazinamide	Fwd	AACAGTTCATCCCGGTTCG	575
		Rev	GTCGCCCGAACGTATGGTG	
rpoB	Rifampicin	Fwd1	GATTCCCGCCAGAGCAAAAC	1281
		Rev1	TAGTCCACCTCAGACGAGGG	
		Fwd2	CCCTCGTCTGAGGTGGACTA	906
		Rev2	GTCGAGCACGTAACTCCCTT	
rrs	Streptomycin	Fwd	TCCAAAGGGAGTGTTTGGGT	1594
		Rev	CAGTTGGGGCGTTTTCGTG	

microorganisms

MDPI

Table S6. Coverage by sequenced resistance regions.

Coverage (resistance regions)			(nreads x reads length/amplicon length)						
	rrs	embB	gyrA	gyrB	inhA	eis	katG	pncA	rpoB
**Sample/amplified length**	**1594**	**3339**	**2588**	**2114**	**825**	**1280**	**2185**	**575**	**2187**
1	1277	11	204	34	2445	1379	988	896	1712
2	5113	29	941	384	11444	5261	6633	29057	10846
3	13470	11	2703	719	19518	8582	7318	5516	15117
4	7484	7	1045	197	10479	6986	5178	4056	10509
5	147	8	23	14	37089	14779	11925	2100	32776
6	14669	4	1243	246	21347	13840	9432	665	18266
7	7101	105	1244	251	10634	6756	5674	43491	9637
8	8801	146	1393	205	16456	7895	9688	0	13179
9	3330	7	275	33	9335	5278	5308	24	25953
10	2749	20	446	52	6077	3609	3003	175831	4459
11	1530	28	273	41	4131	1709	1594	40	2715
12	1463	2	221	35	8100	3442	4409	892	7912
13	446	6	133	6	672	390	335	1857	18610
14	2468	0	141	11	858	137	121	2637	102603
15	3753	25	377	140	5306	3160	3636	17333	6100
16	527	1	5	8	160	5	65	1339	22455
17	1143	9	137	24	3189	1896	974	4223	2297
18	5603	75	910	104	14057	7944	5940	1796	11903
19	7520	38	1466	128	31330	17092	10301	18286	22489
20	874	1	57	10	756	12	69	3083	21432
21	4768	223	1151	349	16081	7098	9274	1624	11306
22	567	3	115	7	699	215	147	2477	30185
23	5	1	2	1	7005	45	7	116578	183
24	789	10	87	2	2383	1111	537	7297	38689
25	274	9	41	1	404	195	96	1028	21205
26	31	7	2	0	2427	52	22	12796	23015
27	6	2	6	1	10	13	3	18	15
28	28	22	33	19	61	56	26	45	81
29	1341	4	5	31	47	12	159	1538	25590
30	732	4	2	1	1976	67	12	7846	12746
31	8	5	7	2	144	31	13	144772	57
32	2008	2	45	32	608	8	181	5346	50573
33	607	1	2	12	54	47	107	1379	19825
34	767	5	2	1	1394	32	151	7022	15031
35	369	5	55	2	560	217	120	1722	14412
36	650	2	100	5	795	306	200	1846	19495
37	289	3	47	2	279	169	102	830	12641
38	4	3	18	1	3670	30	154	888442	166
39	2	1	1	1	58	3	2	7	10
40	1501	2	124	31	1321	17	208	3713	49336
41	249	2	16	4	241	6	47	707	7932
42	606	1	106	2	781	226	91	2673	31688
43	412	2	81	3	527	181	85	1541	17231
44	824	1	82	12	786	51	119	1858	18655
45	1053	2	2	10	100	6	118	2322	31257
46	365	5	11	10	168	9	53	603	11972
47	1158	9	67	4	1960	221	545	8136	9567
H37	850	1	161	4	793	369	183	1849	29213
Mean	2286	18	325	67	5390	2520	2195	32065	17980
SD	3357	41	552	133	8281	4233	3431	131229	17489

Table S7. Coverage by sequenced lineage regions.

Coverage	(nreads x reads length/amplicon length)										
	Lineage 1	Lineage 2	Lineage 3	Lineage 4	Haarlem	LAM	S	X	Lineage 5	Lineage 6	Lineage 7
**Sample/amplified length**	**372**	**356**	**201**	**269**	**320**	**648**	**400**	**374**	**435**	**364**	**212**
1	174	868	238	350	3535	824	609	801	229	772	342
2	1224	5248	3133	2409	22272	2222	2377	3345	1087	3748	2760
3	1726	8318	2399	3156	34521	6668	5019	6290	1650	6665	3189
4	260	5229	1735	2054	22810	4582	3278	4074	239	4307	2128
5	647	18396	4995	7320	82917	8169	8108	12748	657	13087	6669
6	1358	7909	2322	3123	41447	4628	4595	6268	1352	6412	3178
7	2076	5759	1756	2323	19187	5956	4225	6189	2447	6307	2228
8	2496	6016	2446	3174	30906	4149	5025	6650	2420	6237	3059
9	1192	3421	1045	1400	27144	1306	1382	2259	1047	1927	1496
10	808	2341	641	811	9275	1396	1425	1914	856	1720	753
11	37	1759	502	583	4748	1272	898	943	16	1188	516
12	913	2814	990	1148	18359	735	971	1727	897	1604	1462
13	1	1455	1040	701	12	564	323	10	12	933	763
14	6	1753	1029	1233	47	646	699	43	9	1101	763
15	531	1613	687	653	11508	819	632	913	517	916	763
16	18	943	726	650	70	342	93	89	14	32	420
17	373	1275	385	380	3899	938	583	864	459	880	354
18	1967	5352	1120	1539	20963	3770	2728	4159	2170	3932	1563
19	5021	16342	3670	5885	36205	12799	11506	14317	5532	14619	5110
20	1	1896	969	1094	141	539	534	2	16	289	509
21	2934	9737	2319	3086	24336	4247	3683	5923	3144	5679	3410
22	3	2287	1405	1306	152	695	759	8	20	249	919
23	13	48031	2596	14442	59	644	3779	30	9	2105	1768
24	4	5211	3638	2929	23	1036	1361	3	2	1148	2387
25	1	862	510	535	13	292	275	2	2	466	432
26	2	5543	992	2805	2	191	851	7	1	261	611
27	49	715231	6081	81443	2	201	6795	107	37	1395	7015
28	577	2120666	24266	147839	54	4011	18322	505	350	10916	35571
29	6	2236	1514	1418	16	50	29	8	3	6	756
30	17	7238	4677	4212	41	1912	938	2	4	215	3105
31	18	35250	1182	6502	37	25	465	88	16	225	1533
32	6	4896	3208	3292	12	351	153	10	17	19	1710
33	2	1071	649	598	20	330	254	12	27	66	400
34	1	4835	2761	3031	30	896	473	1	20	80	1499
35	4	1566	1091	1032	21	538	552	40	19	120	764
36	11	1900	1482	1197	75	677	689	8	24	213	1040
37	6	830	701	603	411	30	17	32	7	2	463
38	23	71	42	51	203	21	31	28	13	40	104
39	4	126803	2770	20566	119	26	595	18	8	215	2217
40	26	3364	2374	2095	59	1129	978	0	1	306	1563
41	3	471	260	247	94	132	153	7	1	267	211
42	2	2136	1146	1272	57	663	928	6	34	526	753
43	5	1279	872	833	47	495	438	1	7	167	550
44	10	1434	890	949	47	535	617	7	2	668	726
45	1	1191	593	843	11	251	393	18	16	60	339
46	3	508	375	280	1	221	190	1	19	101	230
47	1	8099	5800	4189	15	1812	1929	8	2	1921	3933
H37Rv	3	1750	1207	1025	47	744	298	20	11	47	789
Mean	512	66942	2234	7263	8666	1760	2103	1677	530	2170	2350
SD	991	320060	3556	23912	16115	2517	3354	3226	1063	3423	5143
